# Hand-Holding’s Effect on Children’s Pain Perception and Anxiety during Dental Anesthetic Injections

**DOI:** 10.3390/jcm12216825

**Published:** 2023-10-29

**Authors:** Johnny Kharouba, Gal Berman, Shlomo Elbaharay, Neta Kaplan, Izabella Efremenko, Sigalit Blumer

**Affiliations:** 1 Department of Pediatric Dentistry, The Maurice and Gabriela Goldschlager School of Dental Medicine, The Faculty of Medicine, Tel Aviv University, Tel Aviv 6997801, Israel; galberman9@gmail.com (G.B.); neta.kaplan1989@gmail.com (N.K.); izabellae@mail.tau.ac.il (I.E.); blumer@012.net.il (S.B.); 2 Endodontics Department, The Maurice and Gabriela Goldschlager School of Dental Medicine, The Faculty of Medicine, Tel Aviv University, Tel Aviv 6997801, Israel; shlomodent@gmail.com

**Keywords:** children, pain, anxiety, local anesthetic injection, dentistry, hand-holding

## Abstract

Local anesthetic injections are an essential tool in dentistry, particularly in pediatric dentistry. The needle penetrating the tissue can cause stress, anxiety, and pain. Studies have shown that using touch may alleviate pain and reduce patient anxiety. Yet, this has not been tested in pediatric dental patients. Therefore, this study examined the effect of hand-holding on children undergoing local anesthetic injections. Its effect on children’s pain perception was tested, with the hypothesis that pain perception would be lower for children whose hand was held by an assistant. Additionally, the study examined whether hand-holding would affect children’s anxiety levels and cooperation. Fifty-five children, who underwent dental treatment within the Department of Pediatric Dentistry at Tel Aviv University, were recruited. The patients were randomly divided into two groups. In the study group, the assistant gently placed her hand on the patient’s hand during the anesthetic injection. In the control group, the same treatment was performed without the hand being placed by the assistant. After the anesthetic injection, the child’s pain and anxiety levels were assessed using visual analog scales (VAS). The patients’ pulse was measured. The level of cooperation was evaluated using the “Frankl” scale. Interestingly, although the trends aligned with this study’s hypotheses, no significant effect of hand-holding on pain, anxiety, or cooperation during anesthetic injections was found.

## 1. Introduction

Anxiety, as defined by the American Psychological Association, is characterized by tension, worried thoughts, and physical changes, like increased blood pressure. People with anxiety disorders usually have recurring intrusive thoughts or concerns. They may avoid certain situations out of worry. They may also have physical symptoms, such as sweating, trembling, dizziness, or a rapid heartbeat [1].

Dental anxiety, i.e., anxiety related to dental treatments, is a frequently encountered challenge in dental offices. Dental anxiety evokes an individual’s physical, cognitive, emotional, and behavioral responses. Anxiety is often closely linked to painful stimuli and increased pain perception, and thus, these patients experience more pain that lasts longer. Moreover, they also exaggerate their memory of pain [2].

Many dental procedures can be painful for the patient. Therefore, local anesthetic injections are a prominent tool in dentistry. Local anesthesia is particularly imperative for children who have low self-restraint, and as a result, even small amounts of pain may lead to severe reactions, such as crying and a significant increase in anxiety and stress [3]. Furthermore, when young children experience intense pain, lasting damage to their pain perception may occur [4].

The benefits of using local anesthesia for dental procedures have made it a common practice for both adult and pediatric patients. However, several drawbacks should also be considered. First, the needle penetrating the tissue, along with the administration of the anesthetic, can cause pain. Consequently, the anesthetic injection can also induce anxiety because the anesthetic injection is often the only source of pain during a procedure. Therefore, previous experience leads patients to expect pain caused by the injection. In turn, the expectation of pain increases the level of anxiety caused by the injection [5]. Interestingly, this effect may be bi-directional, as anxiety has been shown to affect pain perception, such that a child experiencing higher anxiety levels may perceive procedures as more painful [6]. The pain caused by the local anesthetic injections can be minimized by optimizing and adapting various factors of the procedure, including the site of injection [7], the pace/rate of injection, and the technique used [8], as well as the experience and skill level of the dentist. Another critical factor that may affect the pain induced by the anesthetic procedure is the devices being used. For example, devices such as DentalVibe, which use vibrations in the region of interest, have been found to alleviate pain and anxiety and boost the patient’s cooperation [9]. A second family of systems is called Computer Controlled Local Anesthesia Delivery System (CCLAD). This includes the Computer Comfort Syringe (CCD), which allows for a controlled rate of injection without a pedal [9], and the popular WAND (Milestone Scientific, Livingston, NJ, USA). This computer-controlled anesthesia supports injections with a constant rate and flow [10]. This approach was also shown to alleviate some of the pain and discomfort caused by the anesthetic injection [8]. Finally, the Jet Injectors (e.g., Med-Jet H-III and Syrijet Mark II) not only alleviate pain but also reduce the tissue damage caused by the injection by leveraging mechanical energy to reduce the pressure caused by fluid entering the tissue [8].

The pain accompanying the local anesthetic injection can be addressed not only by manipulating the injection itself, but also by various other means. Various techniques have been developed to decrease pain levels before administering local anesthetic injections; some are pharmacological, whereas others can be defined as psycho-behavioral. Pharmacological approaches include cooling the tissue with ice and using topical anesthetics. Cooling the tissue with ice or refrigerant sprays induces cryoanesthesia, which acts on all the cells of the area and not just on nerve cells, thereby producing immediate anesthesia, but for a very short time (2–5 s) [11]. Topical anesthetics, such as Lidocaine Patches, Prilocaine, and intra-nasal sprays for front teeth and pre-molar maxillary teeth, are placed on the tissue to anesthetize it slightly before administering local anesthetic injections [8]. Although topical anesthetic is believed to lower the anxiety associated with the injection, it is often only minimally effective in alleviating the injection-derived pain [12]. Moreover, the taste of topical anesthetics is often unpleasant for children [13]. Another pharmacological approach involves the use of nitrous oxide, which relaxes and alleviates the general anxiety levels of the patient, leads to an analgetic effect primarily in soft tissues, and raises the pain threshold such that the pain is perceived as less severe and traumatic [14].

Psychological approaches for alleviating the pain and anxiety associated with local anesthetic injections are frequently used. These often include distracting the patient, techniques from Cognitive Behavioral Therapy (CBT), suggestions, preparation and providing information before the treatment [15], and breath techniques [16]. Other psychological and behavioral approaches include hypnosis, which affects the patient’s pain and anxiety levels [17], using television as a distraction [18], and playing music, though the benefits of the latter require further support [19]. Furthermore, applying various techniques of behavioral management (e.g., de-sensitization, Tell–Show–Do, positive reinforcement, etc.) may encourage the child’s cooperation and lower their anxiety and indirectly lead to, at least partial, pain alleviation [20]. Although there are no contra-indications for using psycho-behavioral techniques, these techniques do not always suffice for alleviating pain and require additional or complementary techniques [21].

A potential approach for pain alleviation during anesthetic injections is touch. Touch is one of the most frequently used methods to sooth children from birth during anesthetic injections [22]. Even in adults, touch has been shown to have a relaxing effect [23]. Additionally, touch was tested on patients undergoing intravitreal injections, where liquid medicine is injected into the space between the retina and the vitreous. Holding patients’ hands mitigated the effect of their anxiety levels before the injection on the pain they perceived [24].

In patients undergoing surgical cataract treatment, it was also shown that hand-holding led to a decrease in both patients’ anxiety and plasma epinephrine levels [25].

Finally, various treatment techniques that stem from the idea that touch is therapeutic (e.g., Reiki) have been shown to affect pain perception [23]. Taken together, these studies suggest that hand-holding carries a positive influence on pain perception for adults, either directly or indirectly, by lowering anxiety. This effect has not been tested in children or studied in the dental domain. 

Hand-holding is a simple and cheap approach to implement, and it could have a wide range of benefits for the treatment, making it a prominent candidate for alleviating pain during local anesthetic injections in pediatric dental care. Therefore, the primary endpoint was to test whether hand-holding during local anesthetic injections in children undergoing dental treatment alleviates their perception of pain and reduces their level of anxiety. The study’s secondary objective was to determine whether hand-holding during local anesthetic injections enhances patient cooperation. 

The study’s alternative hypothesis was that the pain perception level will be lower in children whose hand is being held during local anesthetic injections.

## 2. Materials and Methods

### 2.1. Participants

The research involved 55 children aged 5–15 (with a mean age of 9.79 years, as indicated in Table 1), all classified as American Society of Anesthesiologists (ASA) 1/2. These children underwent a dental procedure that necessitated a local anesthetic injection, with nitrous oxide being the only additional pharmacological anesthetic used. Because of their age, the parents of the participants provided consent by signing a form before their children could participate in the study. 

The study was approved by the University of Tel Aviv Ethics Committee on 10 December 2020 (no. 0002185-2) and was conducted between April and November 2022.

The number of children was predetermined using G*Power3.1.9.4, requiring an accepted power of 0.8, a significance level of 0.05, and after correcting for the ten comparisons that were planned for the study. The study did not include participants who were sensitive to touch, had needle anxiety, were diagnosed with autism, children who refused to have their hand held or who asked for their hand to be held on their own volition, and finally, children that are treated with any kind of sedation other than nitrous oxide.

### 2.2. Study Procedure

The study took place in the Department of Pediatric Dentistry in the school of dentistry at Tel Aviv University. The dentists who treated the children were either dentistry students during pediatric sessions or pediatric dentistry interns.

Twenty-five children were treated at the students’ clinic and thirty children were treated at the clinic of interns. The cooperation of children who were treated by students was positive and definitely positive (Frankel 3 and 4), so they were treated without nitrous oxide. The cooperation of children who were treated by the interns was negative (Frankel 2).

Most of the anesthesia administered by the interns were inferior blocks, whereas the students mostly performed infiltrations.

**Figure 1:**
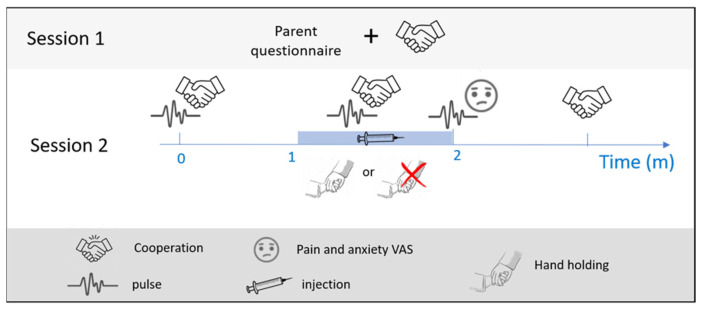
Study protocol. The subjects arrived twice at the dental clinic. The first session (**top**) included a cooperation assessment using the Frankl scale, and the parents filled out questionnaires. The second session (**middle row**) included the treatment. The subjects’ cooperation and pulse were estimated about a minute before the local anesthetic injection was administered and then again during the injection. Immediately after the injection, the subjects’ pulse and self-reports of pain and anxiety were measured one final time. At the end of the treatment, the subjects’ cooperation was estimated once more. Depending on the subject group assignment (control/test), the dental assistant either did or did not hold the subjects’ hand during the injection (symbol legend on the **bottom row**).

The study included two sessions (Figure 1). During session one, the child’s treatment plan was determined, and the parents were briefed about the study and its purpose. The parents were also asked to sign a consent form and complete questionnaires. First, the parents answered demographic questions and questions regarding any previous experience their child had with general and local anesthesia in their dental care. Then, the child’s cooperation was assessed using the Frankl scale [26].

The second session included the treatment, in which the same dentist as in the first session administered the local anesthesia. The children were randomly assigned to two groups using online software (www.randomizer.org) 1 January 2020. One group of children received the anesthetic injection while an assistant was holding their hand (30 children), whereas the other group underwent the same anesthetic injection without hand-holding (25 children). Before the procedure, all children received the same explanation of local anesthetic injections, according to the Tell–Show–Do approach, without a visual presentation of the needle. Furthermore, the procedure also included topical anesthesia (benzocaine), which was applied for one minute for all children. The anesthetic contained Lidocaine 2% with adrenaline 1:100,000 in all cases. The children’s parents/caregivers were present in the room for the whole duration of the treatment.

To assess the effect of touch (i.e., handholding), a student or an additional dentist who was not part of the research team attended the second session and collected various measures. The child’s pulse was acquired using a pulse oximeter (Autocorr, BCI 3304, Smith Medical, Minneapolis, MN, USA) before, during, and two minutes after the local anesthetic injection was administered. Cooperation was estimated using the Frankl scale [26] before the anesthesia, during the anesthesia, and at the end of the treatment. Finally, immediately after receiving the anesthesia, the children were asked to evaluate their pain and anxiety from the treatment using a Visual Analogue Scale (VAS) and Visual Analogue Scale for Anxiety (VASA), respectively. The VAS/VASA presents the children with a scale accompanied by appropriate facial expressions. On one end, the facial expression reflects ‘no anxiety’/‘no pain’, and on the other, ‘high anxiety’/‘high pain’ [27,28].

### 2.3. Statistical Analysis

To test whether hand-holding decreases pain and anxiety during the injection, one-sided unpaired *t*-tests were performed between the handheld and control groups. The VAS scores were used without transformation.

The median resting pulse in normal children decreases drastically between the ages of 5, when it is around 100 bpm, and 15, when it is around 80 bpm [29]. Therefore, we calculated the relative change in pulse from pre-injection to during the injection to estimate the effect on pulse. Hence, the tested variable was as follows: 100 × (pulse during − pulse pre)/pulse pre.

A Mann–Whitney test for cooperation ratings during the injection as a function of hand-holding was performed to test whether hand-holding increased cooperation. Calculations were conducted using Spearman’s correlation between Frankl’s ratings during the injection and Frankl’s ratings at the end of the treatment.

Finally, to test whether the hand-holding effect interacts with or is confounded by the administration of nitrous oxide, a two-way ANOVA test was performed. The test examined the effect of hand-holding, nitrous oxide, and their interaction on pain and anxiety. Similarly, a two-way ANOVA was used to test the interactions between hand-holding and age group on pain and anxiety.

## 3. Results

To test the primary endpoint of this study regarding the perceived pain levels and anxiety among participants as a function of hand-holding during local anesthetic injections, two independent samples *t*-tests, with hand-holding as the independent variable, were performed.

**Figure 2:**
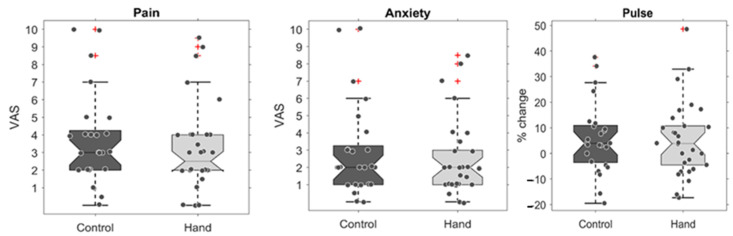
Pain and anxiety with and without hand-holding. The plots show boxplots of the control group (in dark grey) and the test group whose hand was held during the injection (light grey). On each box, the central mark indicates the median, and the bottom and top edges of the box indicate the 25th and 75th percentiles, respectively. The whiskers extend to the most extreme data points that were not considered outliers, and the outliers are plotted individually using the ‘+’ marker symbol. The differences are displayed for the VAS of pain (**left**), anxiety (**middle**), and the relative change in pulse during the injection (**right**). No statistically significant differences were found.

No significant differences were observed for the VAS ratings between the experimental and control groups for pain levels (*t* (53) = 0.65, *p* = 0.259) nor anxiety levels, (*t* (53) = 0.73, *p* = 0.234) (Figure 2).

To complement the objective VAS ratings, further tests were conducted to investigate whether hand-holding led to differences in objective physiological measures. The difference in pulse was tested by comparing the change in pulse from baseline (i.e., pre-injection) to either during the injection or post-injection (Figure 2). No significant differences were found between the groups’ pulse change (during–pre: *t* (53) = 0.45, *p* = 0.326 and post–pre: *t* (53) = 0.91, *p* = 0.181).

**Figure 3:**
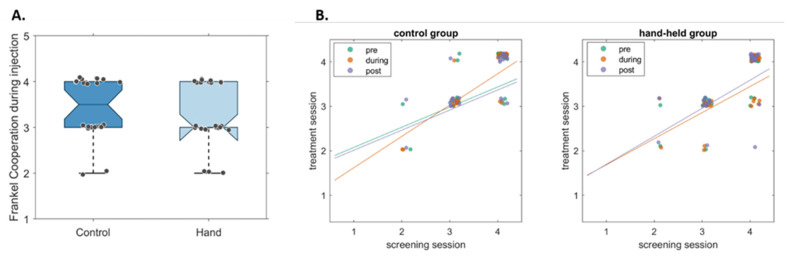
Children’s cooperation at the dental clinic with and without hand-holding. (**A**). Boxplots showing children’s cooperation according to the Frankl scale, both for the control group (in dark blue) and the test group whose hand was held during the injection (light blue). No statistically significant differences were found. (**B**). Cooperation of the control (left) and test (right) groups, calculated pre-injection, during, and post-injection (plotted in green, orange, and purple, respectively), are plotted as a function of their cooperation as determined in a prior session. The lines reflect the linear fits of the data, showing high correlation between the sessions.

To test the second endpoint regarding children’s cooperation as a function of hand-holding, we performed a Mann–Whitney test for cooperation ratings during the injection as a function of hand-holding. No evidence was found for any differences between the control and experimental groups (U = 352, *p* = 0.337) (Figure 3A).

**Figure 4:**
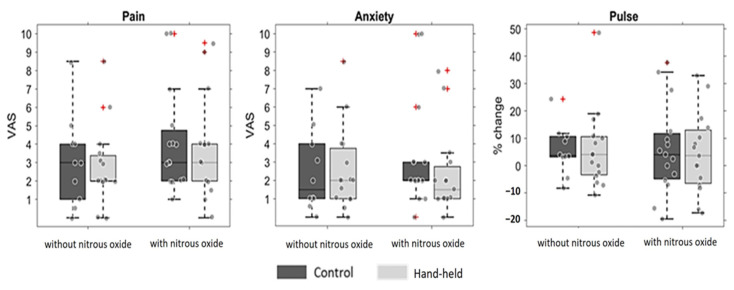
Pain and anxiety levels as reported with and without hand-holding, presented and sorted by nitrous oxide administration. The boxplots show the control group (in dark grey) and the test group whose hand was held during the injection (light grey), separated according to nitrous oxide administration. The whiskers extend to the most extreme data points that were not considered outliers, and the outliers are plotted individually using the ‘+’ marker symbol. The differences are displayed for the VAS of pain (**left**), anxiety (**middle**), and the relative change in pulse during the injection (**right**). No statistically significant differences were found.

To test the confounding effect of nitrous oxide, the test and control groups were separated into two sub-groups/cohorts based on whether they were given nitrous oxide. Thirty children were given nitrous oxide, whereas twenty-five children were not. Figure 4 shows the difference between the groups regarding pain, anxiety, and relative pulse change. The two-way ANOVAs revealed no statistically significant effects of these factors or their interactions.

**Figure 5:**
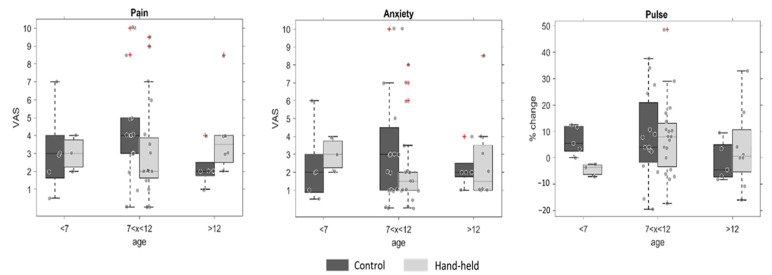
Pain and anxiety with and without hand-holding of the children under dental treatment, classified by age-group. The plots show boxplots of the control group (in dark grey) and the test group whose hand was held during the injection (light grey), separated according to age group. The whiskers extend to the most extreme data points that were not considered outliers, and the outliers are plotted individually using the ‘+’ marker symbol. The differences are displayed for the VAS of pain (**left**), anxiety (**middle**), and the relative change in pulse during the injection (**right**). No statistically significant differences were found.

To test whether age affected the children’s response to touch (i.e., hand-holding), the test and control groups were sorted/separated into three age groups (8 children were under 7 years old, 34 children were between 7 and 12 years old, and 13 children were older than 13 years old). Figure 5 shows the differences between the hand-held and control groups regarding pain, anxiety, and relative pulse change within each age group. A two-way ANOVA revealed no statistically significant effects of these factors or their interactions.

## 4. Discussion

This study tested the benefits of hand-holding in alleviating pain and reducing anxiety in children undergoing local anesthesia for dental treatments. Anesthetic injections are used to eliminate pain during the following treatment but are often a source of pain themselves. As such, anesthetic injections are a common source of anxiety, particularly for children. Existing methods show some efficacy but combining them with psycho-behavioral approaches shows promise in further minimizing children’s pain and anxiety. Studies in adults undergoing non-dental procedures found that hand-holding, as a complementary and cheap approach, was highly beneficial [24]. The present study is the first to test this approach in children and in the dental domain. Interestingly, hand-holding was found to have no effect in terms of pain, anxiety, or cooperation during anesthetic injections. Below, several possible reasons are outlined for the null findings.

The first aim of the present study was to test whether hand-holding during local anesthetic injections in children undergoing dental treatment alleviates their perception of pain and reduces their anxiety levels. VAS ratings were used to estimate the perceived pain levels and found no differences between children in the hand-holding group and those in the control group (Figure 2). Although the differences were not significant, this trend is in line with the research hypothesis (average pain was lower in children whose hand was held during administration of the local anesthetic injection). Since pain perception is subjective and highly variable between children, a between-subject comparison may suffer from low power. Future studies should consider including a within-group comparison, though it is worth noting that, in this case, such a comparison would also be subject to various effects (for example, multiple exposures to the injections could either increase pain perception or lead to adaptation).

Hand-holding was also tested to determine whether it leads to a reduction in anxiety levels during local anesthetic injections. We estimated anxiety using subjective VASA ratings, and like pain perception, although the average anxiety score was lower in the hand-held group, these differences were not statistically significant (Figure 2). As suggested for the pain measurements, these null results could also be partly due to the variability in subjective measurements and the between-subject comparison. Interestingly, this explanation is not sufficient to explain the anxiety results, as objective anxiety estimates were also used, namely the children’s pulse, as an indicator of the physiological response to stress. The pulse measured by the pulse oximeter is a good estimation of heart rate (HR) [29]. Since high HR levels correlate with higher anxiety levels [30], the pulse was used as an additional tool to evaluate the child’s anxiety throughout the treatment.

A possible explanation of this study’s null results, and a potential limitation of the hand-holding approach, is that hand-holding may induce anticipation of a painful stimulus. The children whose hand is being held may expect that the upcoming procedure will be painful, potentially raising their anxiety levels and, consequently, the perceived pain. This process is akin to the nocebo effect, in which negative expectations of a clinical treatment can lead to harmful effects [31]. Future studies may benefit from carefully considering how to incorporate the relaxing effect of touch without inducing negative expectations towards a specific procedure. For example, by holding hands several times throughout the treatment prior to the injection at moments where no pain follows.

The next hypothesis of the study proposed that hand-holding would affect children’s cooperation. Specifically, that hand-holding will improve cooperation during the local anesthetic injection and that cooperation levels during the injection would be a good predictor for the remaining treatment. There were no significant differences between the two groups (Figure 3). This could be partly explained by the stability of this measure. The children participating in this study were very consistent in their cooperation throughout treatment. Children’s cooperation is often shaped by their dental treatment history and experiences. As such, it could be affected, to a lesser degree, by small manipulations within a single procedure. Furthermore, cooperation levels are determined by the treating doctors, and it is possible that, due to cognitive bias, their impression of a child’s cooperation is relatively hard to change. Both could contribute to our finding that the cooperation levels of the children show minimal change, and consequently are not affected by hand-holding.

The effect of hand-holding could potentially interact with other experimental factors. Due to behavior management considerations, some treatments include the use of nitrous oxide, whereas others did not. The use of nitrous oxide is considered highly effective for reducing pain and anxiety. To test whether it may have driven most of the variance in these study measurements, pain and anxiety levels in children were calculated comparing the sub-groups with or without nitrous oxide. Although the interaction was not found to be statistically significant (Figure 4), the study was not designed to answer questions of such interactions, and it is likely that it requires more statistical power to do so. Future studies should repeat this separation into four groups (those with or without handholding and those with or without nitrous oxide) with a larger sample.

Previous studies have found that human touch can alleviate pain and stress when experiencing thermal pain [32] and reduce anxiety during cataract surgery [25]. Crucially, unlike the current study, both studies use adult subjects. Therefore, in the present study, we tested whether the participants’ age might have mediated the effect of hand-holding on their pain and anxiety response to the injection, such that older children would be more responsive to the manipulation. Although no significant interaction between hand-holding and age was found (Figure 5), it should be noted that the oldest children were 15 years old (i.e., not yet adults), and importantly, the majority of children were between 7 and 12 years old. In addition, no significant difference between gender was found (i.e., the effect of hand-holding on boys and on girls). Future studies would benefit from a more balanced age range sample to optimally test the interaction between touch and age.

The present study had several limitations that potentially dampened the effect of hand-holding on pain and anxiety. First, the treatment used two different types of injections (inferior alveolar nerve block and local infiltration), and it is possible that one is more painful than the other. Second, 11 children were treated by dentistry students, whereas the other 44 children were treated by pediatric dentistry interns. The interns were more experienced, which could affect their injection technique and the child’s behavioral management levels compared to students. Moreover, nitrous oxide was not available for student operators, even in cases where it could have been beneficial for the child’s management.

Finally, a unique challenge in the current study was that it was conducted during the covid pandemic. During this time, not only did everyone experience increased levels of anxiety, particularly for events such as dental appointments, but touch was considered a potential source of contagion. As a result, touching strangers was possibly highly anxiety-inducing. Therefore, although hand-holding has been shown to relieve pain and anxiety, and it was emphasized that the hand of the assistant should be placed gently on the child’s hand, it is possible that the fear of catching covid countered this effect. To optimize the positive effect of hand-holding, future studies should consider the identity of the hand-holder. In our study the children’s hand was held by either the operator’s assistant or a non-operating dentist. It may be beneficial to have a parent or familiar caregiver as the one holding the child’s hand. However, hand-holding by the mother can also have a negative effect on anxiety, especially if the mother is anxious. After all, the connection between the mother’s anxiety and the anxiety of the child during dental treatment is well known [33].

This study explored whether hand-holding affects children’s pain perception, anxiety, and cooperation during dental anesthetic injections. Although there were trends consistent with the study’s predictions, the hand-holding intervention did not lead to substantial differences between the groups. For more comprehensive insights, future research should account for the use of other anesthetic aids, like nitrous oxide, and should consider large individual variations in reported experiences. Additionally, subsequent studies might consider having pediatric dentistry interns as the sole practitioners and might consider involving a parent or caregiver for the hand-holding role.

## Figures and Tables

**Table 1:** Demographic data.

Parameter		Participants (N = 55)	Control Group	Hand-Holding Group
Age	Mean ± SD (Years)	9.79 ± 2.5	9.91 + 2.5	9.82 + 2.5
	5–7 years old *n* (%)	8 (14.6%)	5 (20%)	3 (10%)
	8–11 years old, *n* (%)	34 (61.8%)	15 (60%)	19 (63.3%)
	12–15 years old *n* (%)	13 (23.6%)	5 (20%)	8 (26.7%)
Gender, *n* (%)	Males	36 (65.6%)	15 (60%)	21 (70%)
	Females	19 (34.4%)	10 (40%)	9 (30%)
Nitrous oxide, *n* (%)		30 (54.5%)	12 (48%)	18 (60%)
No Nitrous oxide, *n* (%)		25 (45.5%)	13 (52%)	12 (40%)

Abbreviation: SD standard deviation.

## Data Availability

No data is available.

## References

[1] American Psychiatric Association (2013). Diagnostic and Statistical Manual of Mental Disorders: DSM-5.

[2] Appukuttan D.P. (2016). Strategies to manage patients with dental anxiety and dental phobia: Literature review. Clin. Cosmet. Investig. Dent..

[3] Czarnecki M.L., Turner H.N., Collins P.M., Doellman D., Wrona S., Reynolds J. (2011). Procedural pain management: A position statement with clinical practice recommendations. Pain Manag. Nurs..

[4] Mitchell A., Boss B.J. (2002). Adverse effects of pain on the nervous system of newborns and young children: A review of the literature. J Neurosci. Nurs..

[5] Wager T.D. (2005). Expectations and anxiety as mediators of placebo effects in pain. Pain.

[6] Van Wijk A.J., Hoogstraten J. (2009). Anxiety and pain during dental injections. J. Dent..

[7] Kaufman E., Epstein J.B., Naveh E., Gorsky M., Gross A., Cohen G. (2005). A survey of pain, pressure, and discomfort induced by commonly used oral local anesthesia injections. Anesth. Prog..

[8] Kulkarni N., Parakh A., Modi S., Mankare A. (2019). Painless anaesthesia in pediatric dentistry: An updated review. J. Dent. Med. Sci..

[9] Shilpapriya M., Jayanthi M., Reddy V.N., Sakthivel R., Selvaraju G., Vijayakumar P. (2015). Effectiveness of new vibration delivery system on pain associated with injection of local anesthesia in children. J. Indian Soc. Pedod. Prev. Dent..

[10] Kämmerer P.W., Schiegnitz E., Von Haussen T., Shabazfar N., Kämmerer P., Willershausen B., Al-Nawas B., Daubländer M. (2015). Clinical efficacy of a computerised device (STA™) and a pressure syringe (VarioJect INTRA™) for intraligamentary anaesthesia. Eur. J. Dent. Edu..

[11] Lathwal G., Pandit I.K., Gugnani N., Gupta M. (2015). Efficacy of different precooling agents and topical anesthetics on the pain perception during intraoral injection: A comparative clinical study. Int. J. Clin. Ped. Dent..

[12] Parirokh M., Sadeghi A.S., Nakhaee N., Pardakhty A., Abbott P.V., Yosefi M.H. (2012). Effect of topical anesthesia on pain during infiltration injection and success of anesthesia for maxillary central incisors. J. Endod..

[13] Primosch R.E., Rolland-Asensi G. (2001). Comparison of topical EMLA 5% oral adhesive to benzocaine 20% on the pain experienced during palatal anesthetic infiltration in children. Pediatr. Dent..

[14] Kanagasundaram S.A., Lane L.J., Cavalletto B.P., Keneally J.P., Cooper M.G. (2001). Efficacy and safety of nitrous oxide in alleviating pain and anxiety during painful procedures. Arch. Dis. Child..

[15] Sikorova L., Hrazdilova P. (2011). The effect of psychological intervention on perceived pain in children undergoing venipuncture. Biomed. Pap. Med. Fac. Univ. Palacky Olomouc Czech Repub..

[16] Birnie K.A., Noel M., Chambers C.T., Uman L.S., Parker J.A. (2018). Psychological interventions for needle-related procedural pain and distress in children and adolescents. Cochrane Database Syst. Rev..

[17] Abdeshahi S.K., Hashemipour M.A., Mesgarzadeh V., Payam A.S., Monfared A.H. (2013). Effect of hypnosis on induction of local anaesthesia, pain perception, control of haemorrhage and anxiety during extraction of third molars: A case–control study. J. Cranio-Maxillofac. Surg..

[18] Kharouba J., Peretz B., Blumer S. (2020). The effect of television distraction versus Tell-Show-Do as behavioral management techniques in children undergoing dental treatments. Quintessence Int..

[19] Beh W.F., Hashim M.N., Tan W.J., Latiff Z.A. (2018). Music Listening Intervention vs Local Anaesthetic Cream for Pain Management in Infants Undergoing Venepuncture: A Collaborative Trans-Disciplinary Research. J. Pediatr. Res..

[20] Townsend J.A., Wells M.H. (2019). Behavior guidance of the pediatric dental patient. Pediatric Dentistry.

[21] Stack D.M., Bremner G., Fogel A. (2004). The salience of touch and physical contact during infancy: Unravelling some of the mysteries of the somesthetic sense. Blackwell Handbook of Infant Development.

[22] Songer D. (2005). Psychotherapeutic approaches in the treatment of pain. Psychiatry.

[23] So P.S., Jiang J.Y., Qin Y. (2008). Touch therapies for pain relief in adults. Cochrane Database Sys. Rev..

[24] Segal O., Segal-Trivitz Y., Nemet A.Y., Cohen P., Geffen N., Mimouni M. (2016). Anxiety levels and perceived pain intensity during intravitreal injections. Acta Ophthalmol..

[25] Moon J.S., Cho K.S. (2001). The effects of handholding on anxiety in cataract surgery patients under local anaesthesia. J. Adv. Nurs..

[26] Shindova M.P., Belcheva A.B. (2014). Behaviour evaluation scales for pediatric dental patients-review and clinical experience. Folia Med..

[27] Garra G., Singer A.J., Taira B.R., Chohan J., Cardoz H., Chisena E., Thode H.C. (2010). Validation of the Wong-Baker FACES pain rating scale in pediatric emergency department patients. Acad. Emerg. Med..

[28] Hornblow A.R., Kidson M.A. (1976). The visual analogue scale for anxiety: A validation study. Aust. N. Z. J. Psychiatry.

[29] Bloom L.J., Houston B.K., Burish T.G. (1976). An evaluation of finger pulse volume as a psychophysiological measure of anxiety. Psychophysiology.

[30] Billman G.E. (2011). Heart rate variability—A historical perspective. Front. Physiol..

[31] Colloca L., Barsky A.J. (2020). Placebo and nocebo effects. N. Eng. J. Med..

[32] Geva N., Hermoni N., Levy-Tzedek S. (2022). Interaction Matters: The Effect of Touching the Social Robot PARO on Pain and Stress is Stronger When Turned ON vs. OFF. Front. Robot. AI.

[33] Busato P., Rigo Garbin R., Nascimento Santos C., Paranhos L.R., Rigo L. (2017). Influence of maternal anxiety on child anxiety during dental care: Cross-sectional study. Sao Paulo Med. J..

